# The confidante method to measure abortion: implementing a standardized comparative analysis approach across seven contexts

**DOI:** 10.1186/s12963-023-00310-0

**Published:** 2023-07-25

**Authors:** Onikepe O. Owolabi, Margaret Giorgio, Ellie Leong, Elizabeth Sully

**Affiliations:** 1 Vital Strategies, 100 Broadway, 4th Floor, New York City, NY 10005 USA; 2 Guttmacher Institute, 125 Maiden Lane, 7th Floor, New York City, NY 10038 USA

**Keywords:** Confidante method, Abortion measurement, Sub-Saharan Africa, South East Asia, Methodology, Third-party reporting

## Abstract

**Background:**

Obtaining representative abortion incidence estimates is challenging in restrictive contexts. While the confidante method has been increasingly used to collect this data in such settings, there are several biases commonly associated with this method. Further, there are significant variations in how researchers have implemented the method and assessed/adjusted for potential biases, limiting the comparability and interpretation of existing estimates. This study presents a standardized approach to analyzing confidante method data, generates comparable abortion incidence estimates from previously published studies and recommends standards for reporting bias assessments and adjustments for future confidante method studies.

**Methods:**

We used data from previous applications of the confidante method in Côte d’Ivoire, Ethiopia, Ghana, Java (Indonesia), Nigeria, Uganda, and Rajasthan (India). We estimated one-year induced abortion incidence rates for confidantes in each context, attempting to adjust for selection, reporting and transmission bias in a standardized manner.

**Findings:**

In each setting, majority of the foundational confidante method assumptions were violated. Adjusting for transmission bias using self-reported abortions consistently yielded the highest incidence estimates compared with other published approaches. Differences in analytic decisions and bias assessments resulted in the incidence estimates from our standardized analysis varying widely from originally published rates.

**Interpretation:**

We recommend that future studies clearly state which biases were assessed, if associated assumptions were violated, and how violations were adjusted for. This will improve the utility of confidante method estimates for national-level decision making and as inputs for global or regional model-based estimates of abortion.

**Supplementary Information:**

The online version contains supplementary material available at 10.1186/s12963-023-00310-0.

## Background

Representative data on induced abortion are essential to understand the constellation of sexual and reproductive health (SRH) outcomes in a population and assess the degree to which people can exercise their reproductive rights. Unsafe abortion is a leading cause of maternal morbidity and mortality globally [1]. Accurate abortion data can elucidate the conditions under which abortions occur and their subsequent health outcomes. In addition, representative abortion data are required to document the prevalence of unintended pregnancies [2]. These estimates can highlight gaps in contraceptive service provision, aid national governments to better design effective strategies to reduce unsafe abortion, and motivate increased investments in SRH services.

Notwithstanding, obtaining population representative estimates of induced abortion incidence and safety remains challenging due to stigma and legality of abortion [3, 4]. Consequently, indirect estimation approaches are typically applied in countries where official statistics are incomplete or unavailable [4]. One such indirect method that has been increasingly used is the confidante method. It is one of several social network-based methods that exploit third party reporting (TPR) to collect information from respondents on a surrogate sample of women with whom they have reciprocal strong ties [5, 6]. In brief, respondents are asked to think of two or three women they are closest to and report whether each of these women has had an abortion.

In the absence of a gold standard method to measure induced abortion incidence, the confidante method (as with similar TPR approaches) is an attractive methodological option; it has the potential to increase sample sizes of abortions, can be easily added on to reproductive health surveys, and can provide detailed data on the circumstances under which induced abortions occur [6–8]. Thus far, the confidante method has been recently fielded in multiple settings, with published descriptions of study results in Ethiopia, Ghana, India, Indonesia, Côte d’Ivoire, Nigeria and Uganda [9–12].

Despite the growing popularity of the confidante method, research has shown that the method is subject to several potential sources of bias, likely affecting the accuracy of induced abortion incidence estimates [10]. In addition, published papers on the confidante method detail significant variations in how the method was applied and how the analyses were conducted [9–12]. These variations impact the presence of potential biases in the resulting abortion incidence estimates, and limit the comparability of indicators across contexts. For nationally representative estimates of abortion incidence to be used as inputs for global models of pregnancy and abortion [2], potential sources of bias in the application of this method must be addressed in a comparable way. Otherwise, confidante abortion rates could unduly bias model-based estimates, particularly in settings with limited other sources of data on abortion.

A recent publication by Giorgio and Sully 2021 outlines six key assumptions underlying the confidante method, describes how violations to these assumptions may lead to six potential biases study design bias, transmission bias, social desirability/recall bias, selection bias, barrier effects, and popularity bias (see “Appendix A”). Thereafter they proposed methods for identifying and potentially adjusting for these biases during analyses [10]. Within the published confidante method literature, researchers have explored the presence of all biases except popularity bias. However, they have only attempted to adjust for transmission bias and selection bias. Two papers have attempted to adjust for barrier bias using similar approaches and there are no published methods to adjust for popularity bias. The objective of this study is to generate comparable estimates of induced abortion incidence from different contexts using a standardized analytic approach aligned with the conceptual framework in the aforementioned publication. We compare our estimates with previously published results to examine how variations in analytical decisions affected the resulting estimates. Finally, we propose a bias assessment chart that we recommend be included in future publications describing applications of the confidante method. This will help ensure comparability of estimates across contexts and research teams and allow key stakeholders to assess whether resulting estimates are appropriate for influencing policy decisions and service provision, or as inputs for model-based estimates.

## Methods

To identify recent studies using the confidante method, we searched MEDLINE with the terms “abortion incidence” OR “abortion safety” AND “measure*”, for journal articles, observational studies, reviews, or systematic reviews published in any language before June 25, 2020. Out of 40 published studies, we identified seven applications of the confidante method in: Côte d’Ivoire, Ethiopia, Ghana, the island of Java-in Indonesia, Nigeria, Uganda, and Rajasthan state in India [9–12]. Five of the seven studies were fielded on the performance monitoring for action (PMA) survey platform [13]. All surveys were cross-sectional and fielded in 2018. The sampling strategies for each survey were designed to produce nationally representative samples, except for Rajasthan and Java, which were designed to be representative of those sub-national regions. The Additional file 1: Technical Appendix provides additional details about the underlying studies, including their sampling strategies, final sample sizes, measures, and other analytic information not presented in the main body of this paper.

In all applications of this method, respondents are first asked to think of all the women they know who fit the definition of a confidante. While the exact definitions varied across the seven confidante method applications included in this paper (see Additional file 1: Technical Appendix, Table B), they all describe close social ties with whom the respondent shares private information. A key feature of the method is that the confidante definition explicitly states that this relationship must be reciprocal (i.e., confidantes also share private information with the respondent.) In four of the recent applications, respondents could report abortion information on up to three confidantes [10–12]. In the other three applications, respondents were asked to only report on two confidantes [9]. Given the small proportions of women who were able to identify three or more confidantes, we limit our analytic sample to the first and second reported confidantes to ensure comparability.

The core questions for this analysis included the total number of confidantes reported, whether the respondent and confidantes had obtained induced abortions, the month and/or year of the respondent and confidante’s most recent abortion, the degree of certainty respondents had about the induced abortions reported for confidantes (certain and less certain), and whether the respondent had told any of the confidantes about the respondent’s induced abortion experiences (see Additional file 1: Technical Appendix for more details).

## Analysis

We estimate one-year induced abortion incidence rates for confidantes and respondents in each country. For all rates, the numerator includes all abortions that occurred in a specified 12-month time frame. The denominator is the number of respondents or confidantes in the analytic sample. To be included in the confidante rate, respondents had to indicate that they were “certain” that the abortion occurred. We then multiply each rate by 1000 to get the rate per 1000 women of reproductive age (15–49 years) in the corresponding population. Next, we examine the existence of biases across the seven confidante datasets using the confidante method assumptions described in Giorgio et al. When possible, we also attempt to adjust for selection bias, reporting/recall bias, transmission bias in a standardized way across the seven samples*.*

*Selection Bias* One of the most important assumptions of the confidante method is that respondents select confidantes with homophily, which is the principle that a contact between similar people occurs at a higher rate than among dissimilar people [14]. To determine this, we compare the distributions of available sociodemographic characteristics between respondents and their confidantes. In cases where violations of the homophily assumption were identified, confidante incidence estimates were weighted using post-stratification weights created using multiple logistic regression to make the sample representative of the population sampled. (Respondent abortion incidence estimates were weighted using the sample weights generated by PMA or the original study team.) Due to variability in sociodemographic variables collected across contexts and a lack of appropriate auxiliary variables, we were unable to use multiple imputation to construct post-stratification weights for all contexts in a standardized manner. The Additional file 1: Technical Appendix outlines the procedure applied in each context.

We also assess the existence of barrier effects, which would result in study samples missing an important parts of the population [15, 16]. To do this, we used Poisson regression to estimate unadjusted prevalence ratios (uPRs) for the relationship between key respondent sociodemographic characteristics and reporting any (versus no) confidantes.

*Reporting/recall Bias* Given the risk that more recent abortion reporting may be more prone to backward telescoping [17], thereby influencing the validity of the annualized estimates, we compare induced abortion estimates for 2017 (where data were collected for a full year in each context) with annualized estimates for 2018 (where data were collected for a few months in the year). We also compare the 2017 abortion incidence estimates of respondents to their confidantes to check for recall bias.

*Transmission Bias* Previous research notes the importance of accounting for the visibility of abortions when using social-network-based methods to estimate abortion incidence [4, 18]. We apply three methods that attempt to adjust for underreporting due to transmission bias. In one scenario, we included all less certain abortions, regardless of the availability of additional information. In the second scenario, we apply the method detailed by Bell et al. [7] and include less certain abortions where respondents were able to provide additional information about the abortion (where this data was available) in incidence estimates [9]. In the final scenario, we estimate the proportion of respondents self-reporting abortions who shared their experiences with the reported confidantes. Using this information, we apply a correction factor to the base incidence estimates, which is estimated as the inverse of the proportion of respondents who self-reported abortions and had informed any of their confidantes (see Additional file 1: Technical Appendix for a more detailed explanation for the three adjustment methods).

Finally, we conduct a risk of bias assessment on previous publications from each context to examine which assumptions of the confidante method had been evaluated as part of the analysis and the degree of fulfillment or violation of these assumptions. We also compare all confidante adjusted incidence estimates to previously published confidante method estimates from these data to understand how differences in analytic decisions affect resulting incidence rates and other available incidence estimates from the context including recently released country-level estimates from the Bayesian model published by Bearak et al. [19] to understand the relative performance of this method.

## Results

Comparisons between respondent and confidante sociodemographic characteristics are displayed in Table 1. Across all country contexts, respondents did not appear to select confidantes with homophily; there were statistically significant differences in age and education level in all the studies, except for age in Cote D’Ivoire. Generally, the confidante sample was older and more educated than respondents (see Additional file 1: Technical Appendix for distributions of respondent and confidante characteristics.)

**Table 1:** Comparison between respondent and confidante socio-demographic characteristics, by study context

	Cote d'Ivoire	Ethiopia	Ghana	Java	Nigeria	Rajasthan	Uganda
Number of respondents	2738	3668	4596	8969	11,106	5832	2063
Number of confidantes	2024	4062	3731	6680	7836	6030	2727
*Sociodemographic information*							
*Age at last birthday*	*p* = *0.12*	*p* < *0.001*	*p* = *0.026*	*p* < *0.001*	*p* < *0.001*	*p* < *0.001*	*p* < *0.001*
R younger than CF	21.4%	16.2%	20.9%	14.7%	13.3%	10.4%	27.6%
Same age	65.8%	67.8%	63.7%	70.3%	72.7%	72.7%	55.8%
R older than CF	12.8%	16.0%	15.4%	15.0%	14.0%	16.8%	16.6%
*Level of education*	*p* < *0.001*	*p* < *0.001*	*p* < *0.001*	*p* < *0.001*	*p* < *0.001*	*p* < *0.001*	*p* < *0.001*
R less educated than CF	21.9%	19.8%	19.1%	12.7%	14.9%	21.2%	22.4%
Same education	59.4%	62.4%	64.7%	78.1%	73.6%	64.9%	61.0%
R more educated than CF	18.7%	17.9%	16.1%	9.2%	11.5%	13.9%	16.7%
*Place of residence*	*NA*	*p* < *0.001*	*p* < *0.001*	*p* < *0.001*	*NA*	*NA*	*p* = *0.097*
Same location		93.9%	88.8%	91.0%			94.4%
Different location		6.1%	11.2%	9.0%			5.6%
*Marital status*	*NA*	*NA*	*p* = *0.51*	*p* < *0.001*	*NA*	*NA*	*NA*
Both married/cohabiting			51.7%	65.5%			
Neither married/cohabiting			21.6%	17.0%			
R yes, CF no			12.5%	10.6%			
R no, CF yes			14.3%	6.9%			
*Number of children*	*NA*	*NA*	*p* < *0.001*	*NA*	*NA*	*NA*	*NA*
R has less children			22.1%				
Same number of children			53.6%				
More children			24.3%				

*P* values presented here come from Pearson’s chi-square test for independence between the analytic sample of respondents and sample of confidantes in each country. All tests were done using weighted data. The proportions presented in this table represent the distribution of respondent-confidante pairs for each socio-demographic and behavioral indicator

The average number of reported confidantes was less than 1 in four of the seven studies, ranging from 0.79 in Cote D’Ivoire to 1.69 in Uganda (Table 2). Reporting zero confidantes was most common in Java (4374, 48.8%) and Nigeria (5315, 47.0%), and least common in Uganda (404, 19.3%) and Rajasthan (932, 15.8%). Across all studies, there were significant differences in sociodemographic characteristics between respondents who reported any confidantes and respondents who reported none (Table 2). Women with no confidantes were more likely to be older in all contexts, less educated (except Uganda), live in rural areas (except in Cote d’Ivoire, Ghana, and Uganda), be married (except in Ghana and Rajasthan) use family planning (except in Java and Rajasthan) and have more children (except in Uganda).

**Table 2:** Relationships between key respondent sociodemographic characteristics and reporting any confidantes across the seven applications of the confidante method

	Cote d'Ivoire			Ethiopia			Ghana			Indonesia			Nigeria			Rajasthan			Uganda		
	Mean	SD		Mean	SD		Mean	SD		Mean	SD		Mean	SD		Mean	SD		Mean	SD	
Average # confidantes reported	0.79	0.92		1.29	1.11		0.85	0.78		0.89	1.18		0.84	1.26		1.16	1.60		1.69	1.51	
	%	*N*		%	*N*		%	*N*		%	*N*		%	*N*		% N	*N*		%	*N*	
Reported 0 confidantes (%, n)	35.5%	994		25.3%	942		34.6%	1,637		48.8%	4,374		47.0%	5,315		15.8°%	932		19.3%	404	
Reported 1 or more confidantes (%, n)	64.5%	1804		74.7%	2783		65.4%	30,911		51.2%	4595		53.0%	5,988		84.2°%	4983		80.7%	1684	

	uPR	Low CI	High CI	uPR	Low CI	High CI	uPR	Low CI	High CI	uPR	Low CI	High CI	uPR	Low CI	High CI	uPR	Low CI	High CI	uPR	Low CI	High CI
*Age at last birthday*																					
15–19	1			1			1			1			1			1			1		
20–29	0.98	0.91	1.05	0.96	0.92	1.00	1.03	0.97	1.09	0.82	0.78	0.87	1.05	1.00	1.10	1.00	0.97	1.03	1.06	1.00	1.12
30–39	0.98	0.91	1.06	0.83	0.79	0.88	0.95	0.90	1.02	0.66	0.63	0.70	0.95	0.91	1.00	0.96	0.93	0.99	1.01	0.95	1.07
40–49	0.82	0.74	0.91	0.82	0.76	0.87	0.92	0.85	0.99	0.52	0.48	0.55	0.87	0.81	0.92	0.90	0.86	0.93	0.88	0.81	0.96
*Level of education*																					
Never attended	1			1			1			1			1			1			1		
Primary	1.06	0.99	1.14	1.33	1.25	1.40	1.22	1.14	1.32	1.60	1.13	2.25	1.18	1.11	1.26	1.06	1.03	1.10	1.01	0.94	1.08
Secondary	1.15	1.07	1.23	1.42	1.34	1.50	1.35	1.25	1.46	2.54	1.81	3.57	1.31	1.24	1.38	1.06	1.02	1.09	1.08	1.00	1.16
Higher	1.18	1.06	1.32	1.44	1.35	1.53	1.36	1.24	1.50	2.81	2.00	3.96	1.46	1.38	1.55	1.13	1.10	1.16	1.05	0.95	1.16
*Place of residence*																					
Urban	1			1			1			1			1			1			1		
Rural	1.03	0.98	1.09	0.90	0.87	0.94	1.00	0.96	1.04	0.82	0.78	0.85	1.04	1.01	1.08	1.03	1.01	1.06	1.02	0.97	1.07
*Marital status*																					
Married/c ohabi ting	1			1			1			1			1			1			1		
Not married/cohabiting	1.07	1.02	1.14	1.07	1.03	1.11	1.02	0.98	1.07	1.36	1.31	1.42	1.12	1.08	1.16	1.04	1.02	1.07	1.01	0.97	1.06
*Use of any type of family planning*																					
No	1			1			1			1			1			1			1		
Yes*	1.11	1.05	1.18	1.05	1.01	1.09	1.09	1.04	1.14	1.01	0.96	1.06	1.17	1.13	1.22	1.01	0.99	1.03	1.10	1.06	1.15
*Number of children*																					
No child	1			1			1			1			1			1			1		
1–2	0.95	0.89	1.02	0.92	0.88	0.96	1.01	0.96	1.06	0.71	0.68	0.74	0.94	0.90	0.99	0.96	0.94	0.99	1.01	0.96	1.07
3–5	0.92	0.86	0.99	0.82	0.77	0.86	0.95	0.90	1.00	0.62	0.58	0.65	0.88	0.84	0.92	0.94	0.91	0.96	1.00	0.95	1.06
6 +	0.84	0.76	0.92	0.82	0.77	0.88	0.83	0.76	0.90	0.36	0.25	0.51	0.82	0.77	0.87	0.85	0.77	0.92	0.94	0.87	1.00

After comparing the selection bias adjusted 2017 and annualized 2018 abortion incidence rates, we found that the annualized 2018 rate was higher for almost all countries. Due to a concern that more recent reports may be subject to reporting bias [10, 11], we utilize 2017 as the year of reference for annual estimates (see Additional file 1: Technical Appendix, Section C for details and 2018 annualized rates.)

**Fig. 1:**
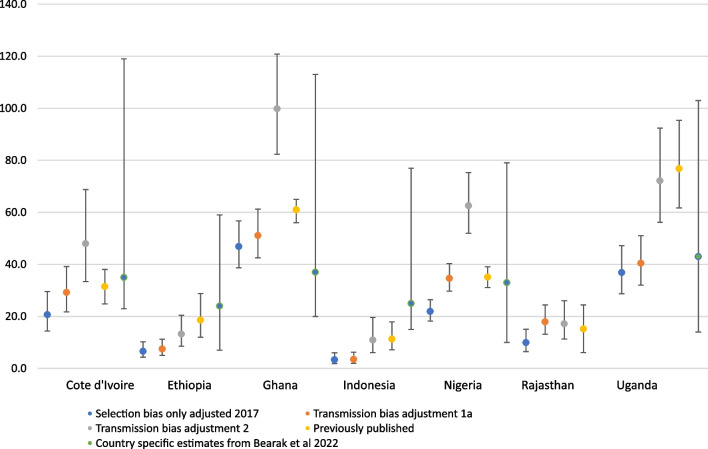
Differences in unadjusted, adjusted and previously published confidante abortion rates, by context

Figure 1 displays five different confidante abortion rates for each country: one-year estimates that are not adjusted for transmission bias (weighted for selection bias), two transmission bias adjusted rates, the published abortion rates from the original studies, and the country-specific rate from Bearak et al.’s Bayesian model [19] (except for Rajasthan which is a state in India and thus did not have a modeled estimate) (More details available in the Additional file 1: Technical Appendix). In the first transmission bias adjustment approach (adjustment 1a), we included all uncertain abortions in the incidence estimate. In Ethiopia, Java, and Uganda, this slightly changes the resulting rates, as few respondents reported they were “uncertain” about their confidantes’ abortions (Additional file 1: Technical Appendix, Table D). Using approach 1b, we included only less certain abortions with additional information on the method used, which was only possible in Cote D’Ivoire, Nigeria and Rajasthan. Across the three countries, there was little to no difference in estimates between the two approaches. As such, we did not include these results in Fig. 1.

**Fig. 2:**
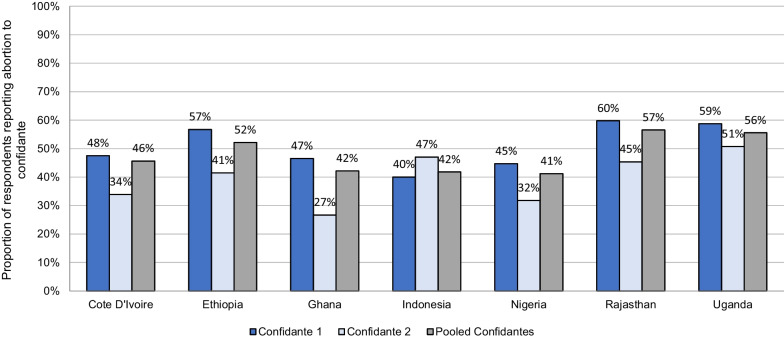
Proportion of respondents who disclosed their abortion information to their confidantes*. *Proportions only calculated among respondents who self-reported their own abortion experiences

In approach 2, we estimated transmission bias using data from respondents who self-reported an induced abortion. This proportion ranged from 0.5% of respondents in Java to 20.1% in Cote D’Ivoire (Additional file 1: Technical Appendix, Table E). Among these respondents, we estimated the proportion who shared this information with their confidantes (Fig. 2). Across pooled confidantes, this ranged from 41% in Nigeria to 57% in Rajasthan. In all contexts except Java, respondents reported their abortions to a higher proportion of confidante 1 compared with confidante 2.

Approach 2 resulted in the highest transmission bias-adjusted estimates; adjusted abortion incidence rates were at least double the non-adjusted rates in all contexts, increasing to an implausibly high rate in Ghana of 99.8 abortions per 1000 women of reproductive age (95% CI 82–121) and very high rates in Nigeria (63.2 per 1000, 95% CI 52–75) and Uganda (72.1 per 1000, 95% CI 56–92).

There is variation between the estimates produced from this analysis and previously published rates (Fig. 1). In Cote D’Ivoire and Nigeria, our adjustment approach 1a rates are comparable to published rates, which is expected given the similarities in the methodologies. However, the transmission bias adjusted rates from this study’s approach 2 are much larger than the previously published estimates in Cote D’Ivoire (48 per 1000 vs. 32 per 1000) and Nigeria (63 per 1000 vs. 35 per 1000). This is likely because the original study only used uncertain abortions to adjust for transmission bias, which does not account for abortions that are completely invisible to respondents. Differences between the rates for Ghana are likely due to this study’s reliance on 2017 reports; the original study noted that the confidante abortion rate from the past 12 months appeared unreasonably high. As such, the one-year confidante abortion rate was annualized using reports of confidante abortions that occurred in the past three years. There was no consistent pattern when comparing the country-specific point estimates from the Bayesian model to our estimates. The modeled point estimates were similar to our approach 2 estimate only in Nigeria, and our approach 1a point estimate only in Uganda.

**Fig. 3:**
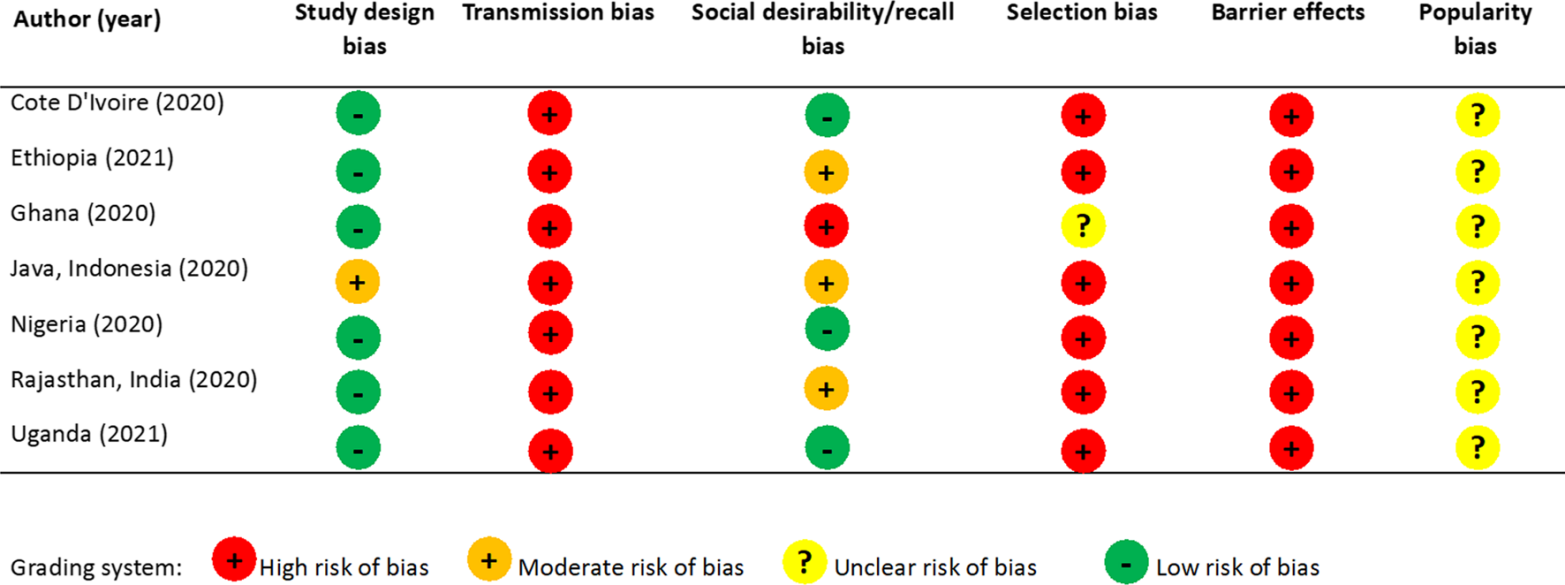
Sample visual chart that can be adapted to summarize biases identified and adjusted for in future confidante studies or reviews of confidante studies

Table 3 shows that majority of the foundational assumptions of confidante method were violated in all seven contexts based on previous publications analyzing this data. Assumptions related to study design and recall bias, which can be assessed by checking for implausibly low confidante abortion rates, were most likely to be met in all contexts except Java and Rajasthan. None of the published papers had attempted to quantify popularity bias (Fig. 3). Selection bias, transmission bias, and barrier effects were most assessed and attempted to be adjusted for in published analyses.

**Table 3:** Risk of bias assessment for studies included in this analysis

	Study design bias	Transmission bias	Social desirability/recall bias	Selection bias	Barrier effects	Popularity bias
Cote D'Ivoire	Assessment status: Assessed, low risk of bias Mode of adjustment: No adjustments made	Assessment status: Assessed, high risk of bias Mode of adjustment: They included confidante abortions that respondents were less certain about if abortion methods were reported. They also imputed confidantes with similar sociodemographic characteristics for confidants who reported zero confidantes and imputed the probability they had obtained abortions in previous years. Thereafter they constructed post-stratification weights to ensure confidante characteristics matched respondents	Assessment status: Assessed, low risk of bias Mode of adjustment: Not adjusted for	Assessment status: Assessed, high risk of bias Mode of adjustment: They included the characteristics of respondents who reported zero confidantes in the confidante one sample and those who reported zero or one confidante in the confidante two sample and applied post-stratification weights to each sample	Assessment status: Assessed, high risk of bias Mode of adjustment: The adjusted for “missing” confidantes by assuming that each respondent who reported zero confidantes has one confidante who shared their characteristics (i.e., assuming perfect homophily). Next, the relationships between reported confidante abortions and other respondent and confidante characteristics are used to predict the probability that each “missing” confidante had an abortion	Not assessed
Ethiopia	Assessment status: Assessed, low risk of bias Mode of adjustment: No adjustments made	Assessment status: Assessed, high risk of bias Mode of adjustment: They calculated a transmission bias adjustment factor that is the inverse of the proportion of respondents who told their confidantes about induced abortion. Then they then apply the confidante-specific adjustment factors to inflate the reported abortions in the past year separately among confidantes. To get the one-year transmission bias-adjusted abortion incidence estimates among all confidantes, they summed the inflated number of abortions by each confidante, divided by the total number of confidantes, and multiplied this estimate by 1000	Assessment status: Assessed, moderate risk of bias Mode of adjustment: Not adjusted for	Assessment status: Assessed, high risk of bias Mode of adjustment: They used multivariable logistic regression to create post-stratification weights so that the confidante samples were nationally representative based on available characteristics	Assessment status: Assessed, high risk of bias Mode of adjustment: Not adjusted for	Not assessed
Ghana	Assessment status: Assessed, low risk of bias Mode of adjustment: No adjustment made	Assessment status: Assessed, high risk of bias Mode of adjustment: Based on an assumption of reciprocity, between respondents and confidantes, they asked respondents who reported an abortion in the past 3 years whether they had disclosed it to each of their confidantes. They calculated the proportion of respondents who disclosed to each confidante, took the inverse of this proportion as the transmission bias factor, computed an average of all three factors (weighted on the number of confidantes) and applied it to the incidence rate adjusted for missing confidantes	Assessment status: Assessed, high risk of bias Mode of adjustment: Given considerable telescoping to the past year and some attrition over time in both approaches, they present direct report and confidante estimates as annualized rates over the past 3 years (mid-2015 to mid-2018)	Assessment status: Assessed, unclear risk of bias Mode of adjustment: They predicted the likelihood of recent abortion for the missing confidante of each confidante-less respondent, using Poisson regression with the respondent’s sociodemographic characteristics as covariates. They then calculated the confidante abortion rates including the missing confidantes’ data	Assessment status: Assessed, high risk of bias Mode of adjustment: They predicted the likelihood of recent abortion for the missing confidante of each confidante-less respondent, using Poisson regression with the respondent’s sociodemographic characteristics as covariates. They then calculated the confidante abortion rates including the missing confidantes’ data	Assessment status: Assessed, Uncertain risk of bias Mode of adjustment: Not adjusted for in analysis
Java, Indonesia	Assessment status: Assessed, moderate risk of bias Mode of adjustment: Not adjusted for	Assessment status: Assessed, high risk of bias Mode of adjustment: They calculated a visibility factor that is the inverse of the proportion of respondents who reported disclosing their own abortion(s) to each confidante. They then applied this visibility factor to the confidante-specific abortion rates to adjust for women’s imperfect knowledge of their confidantes’ abortions	Assessment status: Assessed, moderate risk of bias Mode of adjustment: Not adjusted for	Assessment status: Assessed, high risk of bias Mode of adjustment: They recalculated the community-based survey sample weights so that their respondent sample would more closely match that of the Indonesian Demographic and Health Survey (IDHS). As with the respondents, we calculated adjusted sample weights for the pooled confidantes to match the IDHS sample	Assessment status: Assessed, high risk of bias Mode of adjustment: Not adjusted for	Not assessed
Nigeria	Assessment status: Assessed, low risk of bias Mode of adjustment: No adjustments made	Assessment status: Assessed, high risk of bias Mode of adjustment: They included confidante abortions that respondents were less certain about if abortion methods were reported. They also imputed confidantes with similar sociodemographic characteristics for confidants who reported zero confidantes and imputed the probability they had obtained abortions in previous years. Thereafter they constructed post-stratification weights to ensure confidante characteristics matched respondents	Assessment status: Assessed, low risk of bias Mode of adjustment: Not adjusted for	Assessment status: Assessed, high risk of bias Mode of adjustment: They included the characteristics of respondents who reported zero confidantes in the confidante one sample and those who reported zero or one confidante in the confidante two sample and applied post-stratification weights to each sample	Assessment status: Assessed, high risk of bias Mode of adjustment: The adjusted for “missing” confidantes by assuming that each respondent who reported zero confidantes has one confidante who shared their characteristics (i.e., assuming perfect homophily). Next, the relationships between reported confidante abortions and other respondent and confidante characteristics are used to predict the probability that each “missing” confidante had an abortion	Not assessed
Rajasthan, India	Assessment status: Assessed, low risk of bias Mode of adjustment: No adjustments made	Assessment status: Assessed, high risk of bias Mode of adjustment: They included confidante abortions that respondents were less certain about if abortion methods were reported. They also imputed confidantes with similar sociodemographic characteristics for confidants who reported zero confidantes and imputed the probability they had obtained abortions in previous years. Thereafter they constructed post-stratification weights to ensure confidante characteristics matched respondents	Assessment status: Assessed, moderate risk of bias Mode of adjustment: Not adjusted for	Assessment status: Assessed, high risk of bias Mode of adjustment: They included the characteristics of respondents who reported zero confidantes in the confidante one sample and those who reported zero or one confidante in the confidante two sample and applied post-stratification weights to each sample	Assessment status: Assessed, high risk of bias Mode of adjustment: The adjusted for “missing” confidantes by assuming that each respondent who reported zero confidantes has one confidante who shared their characteristics (i.e., assuming perfect homophily). Next, the relationships between reported confidante abortions and other respondent and confidante characteristics are used to predict the probability that each “missing” confidante had an abortion	Not assessed
Uganda	Assessment status: Assessed, low risk of bias Mode of adjustment: No adjustments made	Assessment status: Assessed, high risk of bias Mode of adjustment: They calculated a transmission bias adjustment factor that is the inverse of the proportion of respondents who told their confidantes about induced abortion. Then they then apply the confidante-specific adjustment factors to inflate the reported abortions in the past year separately among confidantes. To get the one-year transmission bias-adjusted abortion incidence estimates among all confidantes, they summed the inflated number of abortions by each confidante, divided by the total number of confidantes, and multiplied this estimate by 1000	Assessment status: Assessed, low risk of bias Mode of adjustment: Not adjusted for	Assessment status: Assessed, high risk of bias Mode of adjustment: They used multivariable logistic regression to create post-stratification weights so that the confidante samples were nationally representative based on available characteristics	Assessment status: Assessed, high risk of bias Mode of adjustment: Not adjusted for	Not assessed

## Discussion

Despite concerns of the confidante method’s ability to produce reliable estimates of abortion incidence and safety, the method continues to have appeal to researchers due to its ease of implementation and potential benefits. Most importantly, it is one of the only available methods for measuring abortion in some settings, such as informal settlements for refugees or internally displaced persons. Given the likelihood of its continued used, it is essential that researchers appropriately analyze and report confidante data from future studies. This is the first systematic, comparative assessment of the confidante method to estimate the incidence of abortion. We found that variations in the analytic decisions to adjust for existing biases had large impacts on the resulting abortion incidence estimates. This discussion proposes a method for standardizing reporting of confidante data, improving weighting and imputation approaches as one way to account for the lack of homophily, and creating adjustments for transmission bias.

While our study confirms that the confidante method in its current form is severely limited in its ability to accurately measure abortion incidence and safety, other social network-based methods suffer from similar challenges. The network scale-up method (NSUM), which is the most inclusive social network method applied to measure hidden populations, requires population-based data sources for its internal validation making this challenging to apply in many low-income contexts and at-risk populations such as informal urban settlements and humanitarian populations [18]. The Abortion Incidence Complications Method (AICM), which has thus far been the most popular indirect method for measuring abortion incidence, relies heavily on the number of abortion-related hospital admissions and expert opinions, both of which will most likely become less reliable as medication abortion becomes more available. This necessitates further innovation in survey-based indirect approaches to measuring abortion indicators. The confidante method has thus far not been shown to be an improvement over the AICM or other widely used methods, but with a standardized analytical approach and accounting of potential biases, researchers will be better equipped moving forward to evaluate the estimates produced by this method. Our recommendations are as follows:

*Recommendation 1: Future research using the confidante method should clearly report which assumptions were assessed and potentially violated.* For researchers, policy makers, and service providers to appropriately interpret, and/or compare abortion estimates generated through future confidante studies, researchers should document the presence and influence of previously identified biases. If researchers were not able to assess some or all potential biases, this should also be clearly stated. We provide a bias assessment checklist (Additional file 1: Technical Appendix, Table F) that future studies applying this method can include in accompanying papers to document how they attempted to evaluate known biases within their data.

*Recommendation 2: confidante studies should collect more data on confidante’s demographic characteristics to allow the use of rigorous statistical approaches to reweight data.* Across contexts, respondents did not select confidantes with homophily, necessitating that we reweight the confidante sample to the respondents. However, previous studies collected few comparable sociodemographic characteristics for both confidantes and respondents to construct weights using multiple imputation. This likely means that confidante samples remained unrepresentative of the underlying population. That said, it will remain challenging to assess and ensure representativeness if respondents and confidantes differ in unmeasured or unobserved characteristics.

*Recommendation 3: Information on missing confidantes should not be imputed given the systematic differences between respondents who do and do not report confidantes.* Our results suggest that women who reported confidantes differ systematically from those who did not, with over a quarter of respondents reporting no confidantes in five countries. We did not attempt to adjust for barrier effects as had been done in several of the originally published studies [9, 11]. As previously noted, it seemed indefensible to impute such large proportions of the data, given the many assumptions that would need to be met to render this technique appropriate.

*Recommendation 4: Adjustments for abortion visibility should be made using data from respondent’s who self-report abortions.* As expected, there were marked differences between unadjusted incidence rates, transmission bias adjusted rates including less certain abortions, and incidence rates that were adjusted using estimates of abortion visibility. To facilitate clear reporting in future confidante studies, we recommend that publications provide unadjusted “certain” abortion incidence estimates as one indicator. Although this is an underestimate of the true incidence of abortion within a given context, these estimates may be useful as the lower bound data inputs in modeling studies. It is likely that our adjustment 2 approach is more likely to produce a reliable estimate of transmission bias. While including “less certain” abortions is a useful step in attempting to adjust for transmission bias, it is insufficient as it excludes all abortions that are invisible to the respondent Even though the reporting is from a relatively small, non-representative sample of women who self-report abortions, it is also interesting that most of the visibility rates in all contexts in this were close to 50%.

In addition, future studies (particularly those conducted in sub-Saharan Africa) should present incidence estimates separately for abortion and menstrual or period regulation. This will reduce the risk of inappropriately inflating abortion incidence estimates. This is also consistent with previous studies where menstrual regulation to manage early pregnancies is more established legally and culturally [20].

Beyond the recommendations listed above, future research to improve the implementation of the confidante method is needed. One major limitation of the original confidante studies included in this analysis was the use of a narrow definition of a “confidante”. Although these definitions are intended to elicit strong ties and reciprocity in information sharing, it resulted in many respondents reporting zero confidantes and significant differences in respondent characteristics based on whether they reported any confidantes. This likely contributed to the unrepresentativeness of the confidante samples. To ensure that most respondents are contributing to the surrogate sample of confidantes, future studies should test expanded definitions of a confidante that exploits the benefits of strong ties and information while not accidently censoring large proportions of respondents. Another limitation of this analysis was our inability to perform validation tests of the confidante method as was done in the Giorgio and Sully paper using long-acting contraceptive method prevalence rates [10]. Future confidante studies should explore suitable indicators of hidden reproductive behavior to use in validity checks.

## Conclusions

Previous applications of the confidante method have resulted in substantial biases in the resulting incidence estimates. However, given the limited success and applicability of other indirect methods, research should continue to investigate whether the confidante method can be refined in future studies to produce more reliable estimates of abortion incidence. It is important that future improvements to the confidantes and other social network-based methods investigate optimal tie-definitions to enumerate a population representative sample for analysis, collect sufficient data to evaluate the biases associated with these approaches particularly transmission bias, and present their findings using a clear bias assessment checklist. These factors may ultimately affect the utility for confidante estimates both for national-level decision making and as inputs for global or regional model-based estimates.

### Supplementary Information

**Additional file 1: Appendix 1.** Assumptions of the Confidante Method.

## Data Availability

The datasets from India, Côte d’Ivoire, and Nigeria are available in the Performance Monitoring for Action repository (http://www.pmadata.org/) and are available upon request from the host institution. Deidentified versions of the datasets from Ethiopia, Ghana, Indonesia, and Uganda are available from the Guttmacher Institute upon reasonable request to researchers who wish to use the data for scholarly analysis. To discuss obtaining copies of these datasets, please contact popcenter@guttmacher.org with the detailed protocol for your proposed study, and information about the funding and resources you have to carry out the study.

## Appendix A: Assumptions of the confidante method

**Table:**

	Assumption	Biases created by assumption violations	Methods for identifying violations
Assumption 1	Respondents and confidantes share information about their abortions	Study design bias	Investigate whether respondents report any abortions among confidantes Ask respondents who self-report abortions whether they told any of their confidantes about their own abortion Implausibly low confidante abortion rates may be evidence that this assumption may have been violated
Assumption 2	Respondents will have complete knowledge of their confidantes’ abortions	Transmission Bias	Assume this assumption is violated Confirm by asking respondents who self-report abortions whether they shared this information with each of their reported confidantes
Assumption 3	Respondents are willing and able to disclose information on confidantes’ abortions in a survey	Social desirability bias Recall bias	Implausibly low confidante abortion rates are evidence that this assumption may have been violated Compare respondents’ direct reports of abortions to those of abortions among confidantes. If the respondent abortion rate is the same or higher than the confidante rate, then this assumption has been violated
Assumption 4	Respondents select confidantes with homophily	Selection bias	Compare socio-demographic characteristics of the confidante sample to the respondent sample or other nationally representative sample. Systematic differences will indicate that this assumption has been violated
Assumption 5	Respondents who report no confidantes do not differ systematically from respondents who report any confidantes	Selection bias Barrier Effects	Compare key characteristics between respondents who report zero vs. any confidantes. Systematic differences between the two samples will indicate that this assumption has been violated
Assumption 6	Confidante inclusion in the surrogate sample is independent of their abortion status	Selection bias Popularity bias	Difficult to determine Investigate whether respondents were primed to think about abortion prior to being asked to identify confidantes. An overestimate of abortion incidence can also indicate that this assumption was violated

From: Giorgio M, Sully E, Chiu D. An assessment of third-party reporting of close ties to measure sensitive behaviors: the Confidante Method to measure abortion incidence in Ethiopia and Uganda. 2021

## Abbreviations

AICM: Abortion incidence complications method
IC: Informed consent
NSUM: Network scale-up method
PMA: Performance monitoring for action
SRH: Sexual and reproductive health
TPR: Third party reporting
uPRs: Unadjusted prevalence ratios
