# Correction: The confidante method to measure abortion: implementing a standardized comparative analysis approach across seven contexts

**DOI:** 10.1186/s12963-023-00311-z

**Published:** 2023-08-01

**Authors:** Onikepe O. Owolabi, Margaret Giorgio, Ellie Leong, Elizabeth Sully

**Affiliations:** 1 Vital Strategies, 100 Broadway, 4th Floor, New York City, NY 10005 USA; 2 Guttmacher Institute, 125 Maiden Lane, 7th Floor, New York City, NY 10038 USA

**Correction: Population Health Metrics (2023) 21:9** 10.1186/s12963-023-00310-0

The original publication of this article contained an incorrect version of Fig. 1 which was missing the country labels. The revised figure is available in this correction article, and the original article is updated.

**Fig. 1:**
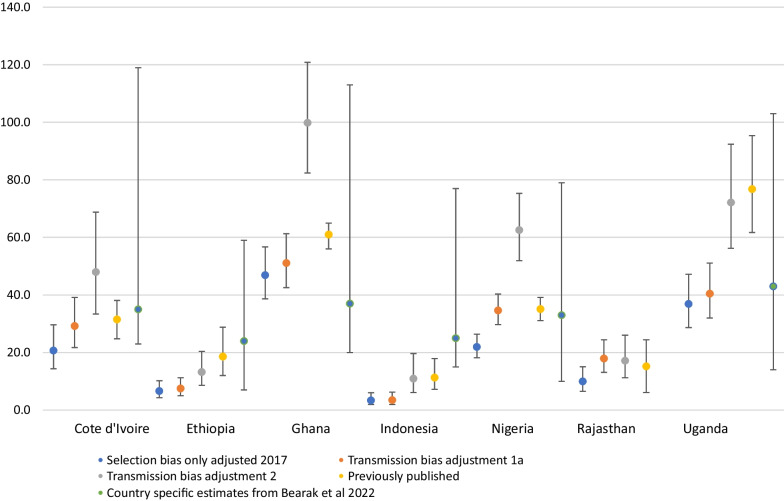
Differences in unadjusted, adjusted and previously published confidante abortion rates, by context

