# Supplementary material for: The confidante method to measure abortion: implementing a standardized comparative analysis approach across seven contexts
Source: Popul Health Metr. 2023 Jul 25;21:9. doi: 10.1186/s12963-023-00310-0 (PMC10369773; doi:10.1186/s12963-023-00310-0)
Supplement: Supplementary file 1 — Additional file 1: Appendix 1. Assumptions of the Confidante Method. [file 12963_2023_310_MOESM1_ESM.docx]

**Technical Appendix**

**Study descriptions**

Although the data for this analysis come from applications of the confidante method in seven different contexts, several of the original studies were related to one another and therefore shared similar approaches. Data from Cote D’Ivoire, Nigeria, and Rajasthan were collected via the PMA platform in each country.[1] The main purpose of those surveys is to measure contraceptive prevalence among women of reproductive age. In 2018, researchers at the Johns Hopkins Bloomberg School of Public Health, in collaboration with the research partners in each country, added the confidante module to measure abortion incidence and safety.[2] As such, the confidante method was applied in a similar manner across those three contexts.

In Ethiopia and Uganda, data were similarly collected via the PMA platform. The Guttmacher Institute, in collaboration with Makerere University School of Public Health, Addis Ababa University, and Johns Hopkins Bloomberg School of Public Health, added several abortion measurement modules to the main family planning survey as part of a study to estimate the impact of the Global Gag Rule on service provision and women’s outcomes in each country.[3–5] Researchers tested two indirect methods in both countries, the confidante approach and the Network Scale-Up Method (NSUM), and respondents were randomized to complete one of the two modules in 2018.[6,7]

In Indonesia and Ghana, data were collected through stand-alone studies whose goals were to test a number of measurement methods for estimating abortion incidence. The Ghana study was designed to be nationally representative and was led by researchers at the Guttmacher Institute, Kwame Nkrumah University of Science and Technology, and Johns Hopkins Bloomberg School of Public Health [8] The study in Indonesia was only representative of the island of Java, which is home to approximately 60% of Indonesia’s population, and was led by researchers at the Guttmacher Institute and the University of Indonesia.[9,10]

**Data Sources and Sample**

In each country, a cross-sectional sample of women of reproductive age (15-49) were selected using a multi-stage cluster sampling design, typically using urban-rural and/or major regions or provinces as the strata. In PMA countries (Cote d’Ivoire, Ethiopia, Rajasthan, Nigeria, and Uganda) and Ghana, a nationally representative (with the exception of Rajasthan, India) sample of enumeration areas (EA) were selected and households within each EA were mapped and randomly selected. A member of each selected household takes part in household survey and all women of reproductive age in the household were interviewed. Similarly in Java, Indonesia, villages were selected from provinces with probabilities proportional to their population sizes and then *Rukun Tetangga* (RTs) were randomly sampled in each selected village. Households in each selected RT were mapped and up to two women of reproductive age were randomly selected from selected households. Once selected, respondents were interviewed in a private location in their home by trained local female interviewers using a structured questionnaire. Data was collected electronically using an Android smartphone enabled with the Open Data Kit (ODK) software.

In PMA countries and Ghana, respondents were dropped from the analytic sample if they only partly completed the household survey or the female questionnaire or reported that they did not sleep at the household the night before the survey. Confidantes identified by those respondents were also subsequently dropped from the analytic sample.

In Ethiopia and Uganda, the confidante method module was randomized to half of the total respondent sample. In this analysis, we analyze respondent data only from those who received the confidante method module.

This leads to the following final samples in each of the countries:

**Table A. Respondent and Confidante sample sizes in each country**

| **Country** | **Number of respondents** | **Number of confidantes** |
| --- | --- | --- |
| Cote d’Ivoire | 2,738 | 2,024 |
| Ethiopia* | 3,668 | 4,062 |
| Ghana | 4,596 | 3,731 |
| Java, Indonesia | 8,969 | 6,680 |
| Nigeria | 11,106 | 7,836 |
| Rajasthan, India | 5,832 | 6,030 |
| Uganda* | 2,063 | 2,727 |

*N’s represent respondents who were randomized to the confidante module, not total sample sizes for the corresponding PMA surveys

**Abortion restrictions in each study context**

Researchers have suggested that the success of social-network based methods in measuring abortion incidence and safety may vary based on the abortion stigma and legal status in a specific context.[11] As such, it is important to consider the legal restrictions on abortion that were in place in each country at the time of data collection. Below we provide the legal classification, as categorized in Bearak et al (2020),(1) as well as a description of the restrictions:

**Cote D’Ivoire: Abortion restricted (permitted only to save a woman’s life)**

Induced abortion is legally restricted unless a pregnancy threatens a woman’s life. The country’s Penal Code states that two medical providers must examine a woman and agree that an abortion is necessary to save her life before a pregnancy can be legally terminated. **Department of Economic and Social Affairs, Population Division, Abortion Policies: A Global Review, Volume 1. Afghanistan to France, New York: United Nations, 2001, pp. 108-109.**

**Ethiopia: Abortion broadly legal**

In 2005, Ethiopia expanded its abortion law, which had previously allowed the procedure only to save the life of a woman or protect her physical health. Abortion is now legal in cases of rape, incest or fetal impairment. In addition, a woman can legally terminate a pregnancy if her life or physical health is in danger, if she has physical or mental disabilities, or if she is a minor who is physically or mentally unprepared for childbirth. **Ethiopia Ministry of Health, Health Sector Development Program IV in Line with GTP, 2010/11–2014/15, Addis Ababa, Ethiopia: Federal Democratic Republic of Ethiopia, 2010.**

**Ghana: Abortion restricted (permitted to preserve health)**

Abortion is a criminal offence regulated by Act 29, section 58 of the Criminal code of 1960, amended by PNDCL 102 of 1985 (1985 amendment to the Criminal Code made abortion legal in cases of rape, incest, fetal abnormality or disease or ‘defilement of a female idiot’ or to protect physical or mental health): **Abortion or miscarriage. Consolidation of Criminal Code of Ghana, 1960. Act 29. Section 58, 10th December 1999; 37-38.**

**Indonesia: Abortion restricted (permitted only to save a woman’s life)**

Current Indonesian abortion law is based on a national health bill passed in 1992: **Health Law 23/1992 (1992)**. Indonesian law allows abortion in medical emergencies, as well as in cases of severe fetal anomaly. In the latter situation, if the woman is married, both she and her husband must consent. The law was expanded in 2009 to legalize abortion in cases of rape, but only up to six weeks’ gestation: **Republic of Indonesia Law on Health No. 36/2009 (2009).**

**Nigeria: Abortion restricted (permitted only to save a woman’s life)**

In Nigeria, abortion is only legal to save a woman’s life.  **Penal Code (Northern States) Federal Provisions Act, Chapter 345 of the Laws of the Federation of Nigeria (Revised ed. 1990), Articles 232-236 Criminal Code Act, Chapter 77 of the Laws of the Federation of Nigeria (Revised ed. 1990), Articles 228-230, 297, 309, 328**

**Rajasthan: Abortion broadly legal**

India’s amended abortion law allows abortion with approval by a medical practitioner under specified conditions that include risk to health and life and sexual assault. Pregnancies up to 20 weeks require authorization by one medical practitioner; and up to 24 weeks authorization by two medical practitioners. In cases involving a severe fetal anomaly there is no gestational age limit, but a three-person medical board, made up of a gynecologist, a pediatrician, and a radiologist, must confirm the diagnosis in order for a pregnant person to access care. **Government of India, The Medical Termination of Pregnancy Act, No. 34, 1971 (amended 2020).**

**Uganda: Abortion restricted (permitted only to save a woman’s life)**

In Uganda, abortion is only legal to save a woman’s life. **Constitution of the Republic of Uganda, 1995, at part XIV(b), XX, articles 27, 34(3) and 41.** The 2006 and 2012 National Policy Guidelines and Service Standards for Sexual and Reproductive Health and Rights provide guidance for the management and prevention of unsafe abortion and highlight several medical indications on which a person can access comprehensive abortion care. The indications highlighted include severe maternal illnesses threatening the health of a pregnant woman e.g. severe cardiac disease, renal disease, severe pre- eclampsia and eclampsia, severe foetal abnormalities which are not compatible with extra-uterine life e.g. molar pregnancy, anencephaly, cervical cancer and HIV-positive women requesting for termination. It argues that these indications fall within the “threat to life” exemption provided by Section 224 of the Penal Code Act. **Ministry of Health. (2012). The National Policy Guidelines and Service Standards for Sexual and Reproductive Health and Rights. Kampala: Republic of Uganda**

**Language used to describe confidantes and abortions**

While all seven studies attempted to measure the same concepts, the language used in each context to describe a confidante and an abortion varied slightly. Some similarities in confidante language between countries is due to the study designs; the surveys in Cote d’Ivoire, Nigeria, and Rajasthan, India were conducted by the same study team at the same time,(2) and the same is true for the surveys in Ethiopia and Uganda.(3) In all studies, discussion with enumerators and study staff during trainings were used to determine the most appropriate terminology for abortion. Table B. displays the exact language used in each survey.

**Table B. Country-specific language used to define confidantes and abortions**

| **Country** | **Confidante Language** | **Abortion Language** |
| --- | --- | --- |
| Cote d’Ivoire | Now I want to ask some questions about your **closest female friends or relatives. These are women whom you share secrets with and who also share theirs with you.** How many female friends or relatives like this do you have in Cote d'Ivoire who are between the ages of 15 and 49? | Do something to remove a pregnancy when you were pregnant or worried you were pregnant  Do something to regulate your period when you were worried you were pregnant |
| Ethiopia | I would like to talk about **women with whom you share intimate secrets and who share intimate secrets with you.** These women might be relatives, like sisters or aunts, or friends. These women should also be people with whom you have been close to for at least a year or more, are 15-49 years of age and live in Ethiopia. We will not ask you to identify the women who you tell us about. How many women do you know that fit this description? | Do anything to intentionally end a pregnancy, in other words induce an abortion |
| Ghana | Currently in your life, think of all the women aged 15-49 who live in Ghana **who would share private and confidential information with you, and with whom you would also share private and confidential information.** These women might be for example relatives, neighbors or friends. They should be people you have been close to for at least a year. We will not ask you to identify these women. How many women do you currently have this type of close relationship with? | Do or use anything to try to end a pregnancy  Do anything to bring on a menses that was late |
| Java, Indonesia | Currently in your life, think of all the women aged 15-49 who live in Java **who would be most likely to share private and confidential information with you that she wouldn't share with everyone, such as sensitive information about her relationships. These must be women with whom you would also share such personal or private information.** These women might be relatives, like sisters or aunts, or they might be neighbors or friends, for example. Roughly, how many women do you currently have this type of close relationship with? | Do something to intentionally end a pregnancy |
| Nigeria | Now I want to ask some questions about your **closest female friends or relatives. These are women whom you share very personal information with and who also share their very personal information with you.** How many female friends or relatives like this do you have in Nigeria who are between the ages of 15 and 49? | Do something to remove a pregnancy when you were pregnant or worried you were pregnant  Do something to regulate your period when you were worried you were pregnant |
| Rajasthan, India | Now I want to ask some questions about your **closest female friends or relatives. These are women whom you share very personal information with and who also share their very personal information with you.** How many female friends or relatives like this do you have in Rajasthan who are between the ages of 15 and 49? | Do something to remove a pregnancy when you were pregnant or worried you were pregnant  Do something to regulate your period when you were worried you were pregnant |
| Uganda | I would like to talk about **women with whom you share intimate secrets and who share intimate secrets with you.** These women might be relatives, like sisters or aunts, or friends. These women should also be people with whom you have been close to for at least a year or more, are 15-49 years of age and live in Uganda. We will not ask you to identify the women who you tell us about. How many women do you know that fit this description? | Do anything to intentionally end a pregnancy |

**Demographic variables**

We identified common demographic questions collected within each country between respondents and confidantes and standardized the construction of these variables across countries. These variables include residence (region/state/province); urban/rural, highest level of education attained (never attended, primary, secondary, higher); age (15-19, 20-29, 30-39, 40-49); parity (no children, 1-2, 3-5, 6+); and current marital status (married/cohabiting, not married/cohabiting).

For comparison of “number of children” between respondents and confidantes in Ghana, “number of children alive” was used rather than “number of children born” as it was used in all other contexts, including comparisons between respondent samples in Ghana.

**Family planning and abortion indicators**

For respondents in each country, the survey instruments measured current use of any type of family planning. We also assessed current use of specific types of family planning for respondents, although this varied depending on the country context (IUD/implant in Cote d’Ivoire, Ethiopia, Ghana, Nigeria, Rajasthan, and Uganda and injectables/pill in Indonesia). Respondents reported if they ever did anything to intentionally end a pregnancy, as well as, which month and/or year this last happened. Respondent abortions were counted if respondents reported that their attempt was successful. The precision of timing of reported respondent abortions varied across countries. In all countries, the year was reported. In Ethiopia, Ghana, and Uganda, month was also reported.

Respondents were also asked if each confidante had ever had an abortion (see Table B for wording), how certain they know this to be true, and when this last happened. Confidante abortions were counted if respondents reported that they were certain about their confidante’s abortion. Like respondents, the precision of timing of reported respondent abortions varied across countries. In Côte d’Ivoire, Ethiopia, Rajasthan, Nigeria, and Uganda, year was reported. In Ethiopia and Uganda, timing was also asked using a pre-coded time frame (less than 1 year ago, 1 to less than 3 years ago, 3 to less than 5 years ago, 5 or more years ago) if respondents were not able to provide an exact year. In Ghana and Indonesia, timing was asked using a relative time frame (x weeks/months/years ago) and a pre-coded time frame. All relative time frames and pre-coded time frames were recoded to exact years based on the date of interview.

In Ghana, a few reported relative time frames for confidante abortions fell ambiguously between December 2017 and January 2018. As such, abortions were counted in 2017 if the date of the interview took place before the 15^th^ of the month and abortions were counted in 2018 if the date of the interview took place on or after the 15^th^ of the month.

In order to compare rates across countries, as well as to understand whether recall bias may be present in respondent’s abortion reports, we calculated two annual abortion incidence rates. The first method calculated the abortion incidence for 2017, as that was the first full calendar year captured in each of the surveys. In cases where respondents did not report a year and were only asked for a relative time frame, we include “one year or less ago” in the 2017 rate. Next, we calculate an annualized rate for 2018. This was done by counting all abortions that were reported for that calendar year, dividing that number by the total number of months that had passed in 2018 at the time of the interview (which raged between 4 in Uganda to 11 in Indonesia), and multiplied that by 12. We then multiplied this by 1,000 to get the rate per 1,000 of reproductive age in each context. Table C displays the differences between the two approaches.

**Table C. Differences between 2017 and annualized 2018 in one-year abortion incidence rates**

|  |  | **Respondent Rates** | | **Confidante Rates** | |
| --- | --- | --- | --- | --- | --- |
|  | **Months of 2018 covered** | **2017 rate** | **Annualized 2018 rate** | **2017 Rate** | **Annualized 2018 rate** |
| Cote D'Ivoire | 7 | 16.86 | 20.83 | 20.69 | 30.88 |
| Ethiopia | 6 | 1.84 | 6.05 | 6.60 | 9.08 |
| Ghana | 6 | 15.59 | 10.85 | 46.87 | 62.25 |
| Indonesia | 11 | 0.27 | 0.56 | 3.36 | 4.36 |
| Nigeria | 4 | 18.72 | 17.90 | 21.94 | 33.39 |
| Rajasthan | 5 | 5.26 | 10.49 | 9.94 | 7.20 |
| Uganda | 4 | 8.47 | 2.34 | 36.88 | 20.83 |

If there were no reporting biases, we would expect that the 2017 and annualized 2018 rates would be similar to one another. However, we find that the 2018 annualized rates are generally higher than the 2017 rates. This could be evidence of recall bias, with respondents being more likely to forget abortions that occurred further back in time. Alternatively, this could be evidence of telescoping, with respondents being more likely to report recent abortions as occurring in the current calendar year, even though they happened earlier to that. Given that we cannot assess which type of bias is present (or whether there was variation in the way biases influenced reporting across country contexts), we chose to standardize our approach by calculating abortion rates for the last full calendar year, which was 2017 in all studies.

**Assessment of homophily**

We used all available confidante socio-demographic characteristics to assess homophily between respondents and confidantes, although the number collected varied by study. Age and education were used in all countries. In Ethiopia and Uganda, residence was also used. In Ghana, residence, marital status, and number of children were used. Lastly, in Indonesia, residence, marital status, current use of any type of family planning, and use of injectables/pill were used.

**Post-stratification weight construction**

After examining differences between the sample of respondents and the sample of confidantes, we determined that the assumption of homophily required for the confidante method was not met in any of the individual countries. We constructed post-stratification weights for each confidante sample based on comparable demographic characteristics with their respective respondent sample to correct this assumption.

In cases where confidante characteristics have missing values, we used multiple imputation with chained equations. We used logistic regression within each imputed dataset and used the pooled estimates to obtain predicted probabilities of being a respondent. To construct analytic weights, we calculated the inverse of the predicted probabilities for each of the confidantes, multiplied it with the female weights, and divided it with its mean. To assess the appropriateness of the regression adjustments, we examined the absolute standardized differences of means and variance ratios for respondent and confidante ages. We also examined weighted differences for each of the categorical variables.

This method was used in the original confidante method analysis in Ethiopia and Uganda and so we elected to import the weights from those datasets for our use. In four of the countries, we determined that multiple imputation was not an applicable method due to a lack of appropriate auxiliary variables. In Côte d’Ivoire, Nigeria, and Rajasthan, we used mean substitution to impute missing data in the confidante sample and proceeded with logistic regression to construct analytic weights as described above. In Indonesia, neither multiple imputation nor single imputation in combination with logistic regression was able to construct appropriate weights for confidantes to match to the respondent sample. As such, we imported the weights created from the original analysis that used respondent values to replace missing confidante values and matched combinations of stratified demographic variables between respondent and confidante samples and the Indonesia Demographic Health Survey (IDHS) sample. After matching on unique combinations of demographic characteristics, the distribution represented by each unique combination in the IDHS sample was divided by the distribution in the respondent and confidante samples to obtain respondent weights and confidante weights.

**Transmission Bias adjustments**

We first use the approach outlined by Bell et al. (2020) for adjusting for transmission bias.(2) In this method, we include less certain abortions for which the respondents could also report the method that the woman used to end her pregnancy. This data was only available in Cote d’Ivoire, Nigeria, and Rajasthan.

In the second approach, we use a less conservative method and simply include all less certain abortions to produce an adjusted estimate. The proportion of all reported confidante abortions that respondents included they were “less certain” about varied by context; uncertainty was highest in Indonesia (45.0%) and lowest in Ethiopia (8.3%).

It is important to note that both the first and second approach only partially adjust for transmission bias. It accounts for abortions that women are not certain about, but it does not account for abortions that are completely invisible to respondents.

**Table D. Proportion of reported confidante abortions that were certain vs. less certain in each context**

|  | **Proportion of abortions reported by certainty** | | | |
| --- | --- | --- | --- | --- |
|  | **Certain confidante abortions** | | **Less certain confidante abortions*** | |
|  | % | n | % | n |
| Côte D'Ivoire | 69.1% | 37 | 30.9% | 22 |
| Ethiopia^ | 91.7% | 45 | 8.3% | 5 |
| Ghana | 89.3% | 158 | 10.7% | 25 |
| Indonesia | 55.0% | 70 | 45.0% | 58 |
| Nigeria | 71.4% | 266 | 28.6% | 122 |
| Rajasthan | 55.4% | 30 | 44.6% | 27 |
| Uganda^ | 89.7% | 116 | 10.3% | 15 |

In the third approach, we estimate transmission bias using information on respondents who reported their own abortions to their confidantes. Respondents with any confidantes who also self-reported an abortion were asked whether they told each of their confidantes about their abortion. We calculated a pooled visibility rate by calculating the proportion of respondents who told either confidante 1 or confidante 2. We then calculated correction factors by taking the inverse of this visibility rate.

**Table E. Visibility of respondent abortions among individual and pooled confidantes**

|  | **Confidante 1** | **Confidante 2** | **Pooled Confidantes** | **Correction Factor** |
| --- | --- | --- | --- | --- |
| Cote D'Ivoire | 0∙48 | 0∙34 | 0∙46 | 2∙19 |
| Ethiopia | 0∙57 | 0∙41 | 0∙52 | 1∙92 |
| Ghana | 0∙47 | 0∙27 | 0∙42 | 2∙37 |
| Indonesia | 0∙40 | 0∙47 | 0∙42 | 2∙39 |
| Nigeria | 0∙45 | 0∙32 | 0∙41 | 2∙43 |
| Rajasthan | 0∙60 | 0∙45 | 0∙57 | 1∙77 |
| Uganda | 0∙59 | 0∙51 | 0∙56 | 1∙80 |

In most contexts, women who self-reported abortions were likely to be older, more educated, live in urban areas, use family planning, have more children and ?? less likely to be married (Data right now in appendix D. Should move here)

**Recommendations for future studies applying the confidante method**

While future studies that use the confidante method to estimate abortion incidence and/or safety should attempt to assess the existence and influence of each of the six biases outlined in Giorgio et al. (2021), this may not be possible in all cases. At a minimum, published reports and manuscripts describing confidante data should include a version of Table F, in which the authors clearly state whether each bias was assessed, how it was assessed, and whether the resulting bias likely had a low, moderate, or high impact on the resulting abortion incidence estimates. Authors may additionally wish to add adjustment techniques used to address these biases if applicable.

**Table F. Template for a risk of bias assessment table for inclusion in Confidante method studies drawing on the assumptions underlying the methods in Giorgio and Sully (2021)**

| **Bias** | **Assessed (Y/N)** | **Mode of assessment** | **Outcome of assessment** |
| --- | --- | --- | --- |
| **Study design bias** |  |  |  |
| **Transmission bias** |  |  |  |
| **Social desirability/recall bias** |  |  |  |
| **Selection bias** |  |  |  |
| **Barrier effects** |  |  |  |
| **Popularity bias** |  |  |  |

**References**

1 PMA. Survey Methodology | PMA2020. Perform. Monit. Account. 2020. 2018.https://www.pma2020.org/survey-methodology (accessed 30 May 2018).

2 Bell SO, Shankar M, Omoluabi E, *et al.* Social network-based measurement of abortion incidence: promising findings from population-based surveys in Nigeria, Cote d’Ivoire, and Rajasthan, India. *Popul Health Metr* 2020;**18**:28. doi:10.1186/s12963-020-00235-y

3 Giorgio M, Makumbi F, Kibira SPS, *et al.* Investigating the early impact of the Trump Administration’s Global Gag Rule on sexual and reproductive health service delivery in Uganda. *PLOS ONE* 2020;**15**:e0231960. doi:10.1371/journal.pone.0231960

4 Sully E, Shirefaw S, Seme A, *et al.* Impact of the Trump Administration’s expanded Global Gag Rule policy on family planning service provision in Ethiopia. under review.

5 Giorgio M, Makumbi F, Kibira SPS, *et al.* An investigation of the impact of the global gag rule on women’s sexual and reproductive health outcomes in Uganda. *Rev* 2021.

6 Giorgio M, Sully E, Chiu DW. Rethinking the appropriateness of third party reporting of close ties to measure abortion: an assessment of the Confidante Method for estimating abortion incidence in Ethiopia and Uganda. under review.

7 Sully E, Giorgio M, Anjur-Dietrich S. Estimating abortion incidence using the network scale-up method. *Demogr Res* 2020;**43**:1651–84. doi:10.4054/DemRes.2020.43.56

8 Keogh SC, Otupiri E, Chiu DW, *et al.* Estimating the incidence of abortion: a comparison of five approaches in Ghana. *BMJ Glob Health* 2020;**5**:e002129. doi:10.1136/bmjgh-2019-002129

9 Giorgio M. Incidence of Induced Abortion in Java, Indonesia, 2018. *Int Perspect Sex Reprod Health* 2020;**46**.

10 Stillman M, Leong E, Utomo B, *et al.* An Application of the Confidante Method to Estimate Induced Abortion Incidence in Java, Indonesia. *Int Perspect Sex Reprod Health* 2020;**46**:199–210. doi:10.1363/46e0120

11 Rossier C, Feehan D, Owolabi O, *et al.* A multiplex RDS for abortion? Assessing the potential of the Respondent-Driven Sampling to study abortion safety in restrictive contexts. 2018.
